# Assessing species richness trends: Declines of bees and bumblebees in the Netherlands since 1945

**DOI:** 10.1002/ece3.5717

**Published:** 2019-11-07

**Authors:** Tom J. M. Van Dooren

**Affiliations:** ^1^ CNRS ‐ UMR 7618 Institute of Ecology and Environmental Sciences (iEES) Paris Sorbonne University Paris France; ^2^ Naturalis Biodiversity Center Leiden The Netherlands

**Keywords:** nonlinear generalized linear model, pollinator trends, Pradel model, sampling heterogeneity, species richness

## Abstract

Estimating and predicting temporal trends in species richness is of general importance, but notably difficult because detection probabilities of species are imperfect and many datasets were collected in an opportunistic manner. We need to improve our capabilities to assess richness trends using datasets collected in unstandardized procedures with potential collection bias. Two methods are proposed and applied to estimate richness change, which both incorporate models for sampling effects and detection probability: (a) nonlinear species accumulation curves with an error variance model and (b) Pradel capture–recapture models. The methods are used to assess nationwide temporal trends (1945–2018) in the species richness of wild bees in the Netherlands. Previously, a decelerating decline in wild bee species richness was inferred for part of this dataset. Among the species accumulation curves, those with nonconstant changes in species richness are preferred. However, when analyzing data subsets, constant changes became selected for non‐*Bombus* bees (for samples in collections) and bumblebees (for spatial grid cells sampled in three periods). Smaller richness declines are predicted for non‐*Bombus* bees than bumblebees. However, when relative losses are calculated from confidence intervals limits, they overlap and touch zero loss. Capture–recapture analysis applied to species encounter histories infers a constant colonization rate per year and constant local species survival for bumblebees and other bees. This approach predicts a 6% reduction in non‐*Bombus* species richness from 1945 to 2018 and a significant 19% reduction for bumblebees. Statistical modeling to detect species richness time trends should be systematically complemented with model checking and simulations to interpret the results. Data inspection, assessing model selection bias, and comparisons of trends in data subsets were essential model checking strategies in this analysis. Opportunistic data will not satisfy the assumptions of most models and this should be kept in mind throughout.

## Graphical abstract

Two methods are proposed and applied to estimate richness change. The methods are used to assess nationwide temporal trends (1945–2018) in the species richness of wild bees in the Netherlands.

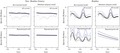

## 1 INTRODUCTION

Species richness, the number of species present in a community or assemblage, is an important component of biodiversity. Methods for estimating species richness have been given a lot of attention (Gotelli & Colwell, 2011). In particular, obtaining local estimates of species richness by means of multispecies occupancy modeling (Dorazio & Royle, 2005; Guillera‐Arroita, Kéry, & Lahoz‐Monfort, 2019) has seen a recent surge in interest. In comparison to such estimation of local occupancy and the estimation of sizes of local and total species pools, assessing species richness changes over time seems to have received less attention in method development. There seems to be no general strategy for comparing more than two samples or for estimating a time trend when not all species have been sampled or detected. For data collected in an opportunistic manner without standardized protocols or a planned sampling scheme, even the analysis of a trend in a single focal species is not straightforward (Isaac, Strien, August, Zeeuw, & Roy, 2014).

However, this contrasts with the need to obtain adequate trend estimates even when only opportunistic data are available. For example, in the face of climate change and when accounting for geographical crop variation, the conservation of pollinator richness levels might be crucial to mitigate effects of ecosystem change (González‐Varo et al., 2013; Klein, Müller, Hoehn, & Kremen, 2009). It also seems that an overall and substantial decline in total biomass, such as observed for insects in nature reserves in Germany (Hallmann et al., 2017), will reduce species richness inevitably. Species richness trends of pollinators providing essential ecosystem services therefore merit a lot of attention and different drivers of pollinator abundance and richness change have already been identified (Potts, Imperatriz‐Fonseca, Ngo, Aizen, et al., 2016; Potts, Imperatriz‐Fonseca, Ngo, Biesmeijer, et al., 2016). Marshall et al. (2018) stated that bumblebees in Europe have been in a steady decline for decades. However, in a recent analysis of species richness trends, Carvalheiro et al. (2013) concluded that declines in species richness have slowed down for several taxa in NW‐Europe, including bumblebees. A reassessment of that analysis (Van Dooren, 2016) concluded that it only provided support for decelerating declines for the bees *Anthophila* in the Netherlands, conditional on accepting the inference strategy and parameter estimates as valid. An inspection of the scripts executing the analysis of Carvalheiro et al. (2013) found that many of their estimates have uncorrected errors or lead to anticonservative inference (Van Dooren, 2016).

The Dutch wild bee data in these studies are a heterogeneous mix: the records (sensu Isaac & Pocock, 2015) were collected in different ways (observations, observations submitted to a website, samples deposited in collections, by hundreds of observers and collectors) and relatively unplanned. This might be exemplary for most data collected in citizen science efforts. However, this heterogeneity was unaccounted for in previous analyses. In Carvalheiro et al. (2013), data were binned in arbitrary time periods while a year‐to‐year continuous time analysis would be insightful and could provide more detail on patterns of change, in particular on whether decelerating declines occur. For the reanalysis, I chose to reconsider the inference strategy. It applies two promising approaches for assessing species richness trends among four explored (Appendices S1–S3) and compares their merits, assisted by simulations. The first analyses time patterns in species richness by modeling time‐dependent asymptotes of species accumulation curves. In an alternative statistical analysis, richness will not be estimated itself for reasons detailed below. There, time patterns in the rates of colonization and local (i.e., within the Netherlands) extinction will be estimated. Both assume nonexhaustive sampling of the species present and assess richness trends at the level of the entire Netherlands. The opportunistic nature of the data implies the absence of any spatial sampling scheme or design in the data. I therefore decided not to attempt to assess important local variability in richness trends (Yoccoz, Nichols, & Boulinier, 2001).

## 2 MATERIAL AND METHODS

Species richness is the horizontal asymptote of a species accumulation curve representing the expected number of species in a sample as a function of sample size. Estimators of this asymptote are often biased (Walther & Moore, 2005), or the precision of an estimate can be limited (O'Hara, 2005). In a comparison of two assemblages, species accumulation curves can cross (Chao & Jost, 2012; Lande, DeVries, & Walla, 2000; Thompson & Withers, 2003; Van Dooren, 2016), such that patterns in the relative numbers of species found at low sampling efforts or at rarefied sample sizes (e.g., Biesmeijer et al., 2006) can be independent of actual species richness differences.

Gwinn, Allen, Bonvechio, Hoyer, and Beesley (2016) found high sensitivity of bias and precision of change estimates to the relative abundances of species in a dataset. Gonzalez et al. (2016) argued that the risk of biased estimates of change needs to be considered in short time series of species richness. Similarly, Cardinale, Gonzalez, Allington, and Loreau (2018) drew attention to risks of using estimates of the earliest time point as baseline, pointing to the possibility of mistaking regressions to the mean for actual responses. Additionally, nonlinear data transformations can in particular affect the impression time series give us, as the distance between data points will change more in some intervals due to the transformation than in others.

### 2.1 Data

**Figure 1:**
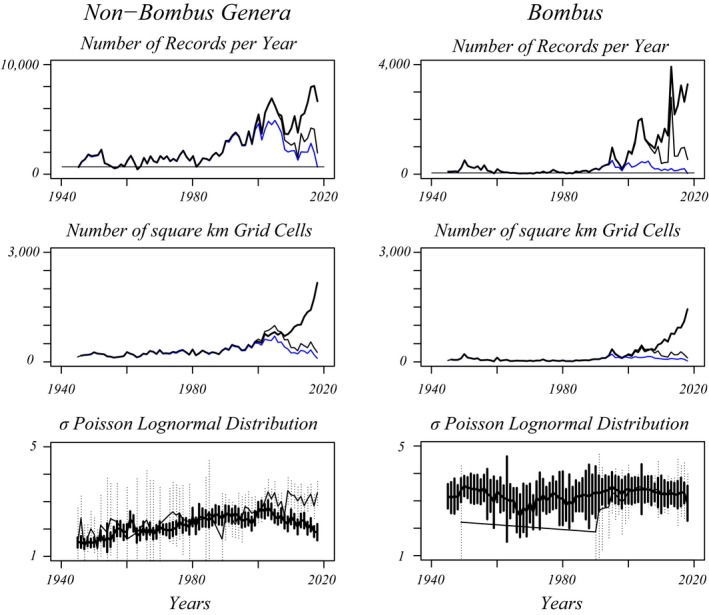
Sampling strategy and volume of wild bee data in the Netherlands have changed over the years. Top row. Total numbers of records per year have changed substantially over the period (thick black lines). Note the difference in scale between both panels. The fractions of records that represent individuals deposited in collections have become a minor fraction of the totals in recent years (thin blue line). The difference is caused by an increasing number of observations without specimens deposited in collections, of which the fraction contributed by volunteers on the website https://waarneming.nl is increasing (thin black line: all records except those contributed through https://waarneming.nl). Horizontal lines are drawn at twice the total number of species sampled over the entire period per taxonomic group. For points below this line, there are on average fewer than two observations per species. Middle row. Records are obtained in a variable number of square kilometer grid cells per year (thick: total numbers, thin blue line: samples deposited in collections, thin black: samples in collections plus observations not contributed via https://waarneming.nl). Bottom row. When distributions of records per species per year are analyzed using Poisson lognormal models (Engen et al., 2002), the *σ* heterogeneity parameter for their distribution shows a gradual change over the years (estimates for different years connected by a line, approximate 95% confidence intervals for each estimate. Thick/thick: samples deposited in collections, thin/dotted: observations)

The dataset analyzed here consists of 73 years of opportunistic data. Bee records in the EIS (European Invertebrate Survey Netherlands) database in the period 1945–2018 are analyzed, extending periods investigated in Biesmeijer et al. (2006), Carvalheiro et al. (2013) and Van Dooren (2017). The data were provided by EIS as a list of records with species names, years of collection, and the square kilometer cell where each record was collected. The dataset analyzed in Van Dooren (2017) turned out to contain 150,047 spurious records, presumably due to a query error and therefore had to be redone. Sampling has not occurred in a standardized manner across the study period, and not in a spatially homogeneous or balanced manner either (Figure 1). In particular from mid‐eighties until around 2005, increasingly larger numbers of records have been collected, more often by observation and with records from a larger number of square kilometer grid cells (Figure 1). For both non‐*Bombus* and *Bombus* bees, there have been years where the number of records was less than twice the number of species sampled over the entire study period (four, resp. eleven times). In particular for bumblebees, 1966–1971 and 1981–1983 were periods of consecutive years with such relatively low sampling intensities. These years are expected to contribute little to the estimation of species richness and indicate that insufficient data are available for an analysis at finer spatial scales. The observation data contributed by volunteers through the website https://waarneming.nl since 2005 is from a steadily increasing number of grid cells per year (Figure 1), such that the number of observations per grid cell with records now steadily decreases, again complicating any analysis at smaller spatial scales. The samples per year of non‐*Bombus* species show important time trends in the heterogeneity parameter *σ* of Poisson lognormal distributions (Engen, Lande, Walla, & DeVries, 2002). This parameter *σ* is a compound measure representing abundance variation between species rescaled by species‐specific sampling effort if present. Its changes suggest that the heterogeneity in numbers of individuals per species in the samples has increased during the study period.

I assessed patterns of species richness change over time in the entire Netherlands and therefore fitted models with explanatory variables that changed over time to all data or data subsets that in principle span the entire Netherlands. Previous conclusions were drawn at that scale and I expect that changes at smaller scales are increasingly affected by (nonrandom) sampling variability and lack of consistent protocol. The models fitted did not contain any parameters or variables representing spatial species richness differences or heterogeneity. Only the number of grid cells sampled was added as an explanatory variable because it can be expected to affect predicted nationwide trends. To facilitate comparisons with Carvalheiro et al. (2013), time patterns in species richness of *Bombus* and non‐*Bombus* bee genera in the EIS database were analyzed separately. The data were analyzed in different ways which should be seen as separate attempts to arrive at a satisfactory model for richness change. I repeat the statement of Guillera‐Arroita et al. (2019) here: “we cannot model our way out of every situation,” which I believe to apply in particular to situations with opportunistic data.

Each approach accounts for imperfect detection of species, which should never be ignored (Guillera‐Arroita, 2017) and for time trends in sampling strategy. Within each approach, model simplification is carried out with the purpose of obtaining the best possible predictions without a priori assuming that the true model is among the candidates compared, hence by means of AICc comparisons (Akaike information criterion adjusted for finite sample sizes, Burnham & Anderson, 2002) as AIC(c) model selection is efficient (Claeskens & Hjort, 2008). Model selection, parameter estimation, and inference in general were backed up as follows. Next, to an analysis of all data, two subsets of records were analyzed separately and for each method: (a) Records for which the individual was deposited in a museum collection, excluding for example observations. (b) Records collected in square kilometer grid cells that were sampled in three periods of approximately equal length. These are the last period where the number of observations and grid cells have drastically increased (1994–2018; Figure 1), a period of equal length right after the end of WWII (1945–1969) and the years in between (1970–1993). In this subset, spatial locations that contributed samples in a restricted time period only were excluded. This avoided that in one part of the study period richness might have been assessed for a different set of grid cells than in another.

To the entire dataset and to the subsets, different models were fitted. Model simplification increases the precision of the remaining individual parameter estimates, but this can come at the cost of larger estimation bias. In each statistical analysis presented below, I assessed effects of this bias‐variance trade‐off (Claeskens & Hjort, 2008) by comparing the predicted time trend of species richness between the best maximal model fitted and a simplified minimum adequate model (see below). I will only conclude that a deceleration occurs when it is detected in the full data and in the data subsets and does not suddenly emerge as a result of model selection bias. To understand patterns of estimation bias, the analysis was further complemented with simulations.

### 2.2 Generalized nonlinear models

In a first approach, the number of records and the number of species per year in a dataset were used to estimate nonlinear species accumulation curves (Gotelli & Colwell, 2011, Appendix S3) with parameters that change over time. In other studies, nonlinear power functions without horizontal asymptote have been fitted (Ugland, Gray, & Ellingsen, 2003) that predict species richness by extrapolating from a smaller area to a larger fixed range. Here, species accumulation curves are represented by Michaelis–Menten curves *a*(*t*)*x*(*t*)/(*b*(*t*) + *x*(*t*)), estimating species number with two time‐dependent non‐negative functions *a*(*t*) and *b*(*t*) and with *x*(*t*) the number of records in year *t*, representing sample size. Function *a*(*t*) is the time‐dependent horizontal asymptote of the curve, hence it is this part of the model which describes the change in species richness over time. I assume that functions *a*(*t*) and *b*(*t*) change gradually across years and therefore used smooth functions (Wood, 2006) to parameterize them and assess their gradual changes. We can expect that *b*, which quantifies the increase in species number with sampling effort shows a time pattern affected by changes in sampling method and the number of grid cells sampled. The ratio *b*/*a* is an estimate of the Simpson diversity in random samples (Lande et al., 2000). If species richness, given by the asymptote *a*, has a decelerating decline, we should be able to observe that in the predicted time pattern for *a*(*t*). Note that this model representing a species accumulation curve combines the estimation of state variable *a*(*t*) with properties of the observation/detection process captured by function *b*(*t*). This approach does not need specification of a distribution of capture probabilities per species, nor a distribution of species abundances (e.g., O'Hara, 2005 for some examples).

Michaelis–Menten curves were fitted to numbers of species sampled per year. I used the function *gnlr*() in R (Lindsey, 1997) which maximizes the model likelihood using a general purpose optimizer. None of the estimation methods proposed by Raaijmakers (1987) nor the nonlinear least squares used by O'Hara (2005) were used, hence the performance assessments of O'Hara (2005) and Walther and Moore (2005) of this estimation method do not immediately apply. Functions *a* and *b* in the model were fitted with natural cubic splines of the year variable of up to eight degrees of freedom and exponential link functions assuring non‐negativity of *a* and *b*. Gaussian errors were assumed (Colwell et al., 2012) with variances specific to each year. In simple tests for a change in species richness, Gwinn et al. (2016) found inflated type I errors when using estimates of species richness as data. To limit this, the actual species counts are used as data, and their variances are modeled and kept above a minimum. The error variance in the model consisted of the sum of an estimated variance parameter plus an offset equal to the unconditional sampling variance of the number of observed species in a year given multinomial sampling of individuals (equation 5 in Colwell et al., 2012). The variance regression equation ensured that the error variance never became smaller than this multinomial sampling variance (Appendix S3 gives an example of the code). This is reasonable given that we do not know how much the sampling was different from random, which is an extra source of uncertainty. For the curvature function *b*(*t*), alternative models were fitted with splines (up to eight *df*) of the total number of grid cells with data per year or of the *σ* parameter of the Poisson lognormal distribution (Engen et al., 2002) fitted to the data per year for the group analyzed. Model simplification occurred in a manner which slightly reduced the total number of models to fit and compare. I fitted all models with splines of 8, 5, 3, and 1 degrees of freedom and the model with no explanatory variables for *a* or *b*. Among the models in this set, I selected the one with the lowest AICc. Then, models with one *df* added to each spline in this model or with one *df* removed were also fitted, to check whether these modifications would reduce the AICc further. The model with the lowest AICc among all the ones fitted is called the "minimum adequate” model. This model is compared with the “maximal” model: the model with lowest AICc among those with splines of 8 *df* for both *a*(*t*) and *b*(*t*).

A bootstrap estimate of estimation bias (Davison & Hinkley, 1997) was obtained as follows. One hundred bootstrap pseudodatasets were constructed by randomly drawing for each year a number of individuals equal to the number of records in that year and repeating the analysis above for each of these sets of pseudodata. The covariates calculated from the original data were kept except for the multinomial sampling variances which were recalculated for each sample. The maximal and adequate models were fitted to each of these datasets, and species richnesses per year predicted. The estimated bootstrap bias equaled the average of these predictions per year minus the predicted value obtained from the original data. Note that this straightforward bootstrap differs from the one proposed by Chao et al. (2014), in that the number of undetected species is not estimated and incorporated in the resampling.

### 2.3 Capture–recapture analysis of species encounter histories

Given the recent interest in occupancy methods and the possibility they offer to estimate local and total species richness (Kéry & Royle, 2008), it needs to be argued why these methods were not used here. Many implementations of occupancy models assume no changes in total richness (closedness). This does not prevent assessing richness trends in itself. For example, Dennis et al. (2017) first estimated occupancy per species year assuming closedness and used a weighted regression on these single‐species estimates to test for trends over time. Repeated observations within years are required at a fixed set of locations for estimating occupancy, which does not match with the structure of the bee data and the lack of data in some years. van Strien et al. (2016) constructed detection/nondetection records per square kilometer cell with observations and analyzed these per species separately. In this dataset, there is no guarantee that collectors sampled in a manner representative of the species locally present and I therefore refrain from such reconstructions.

Furthermore, while Kéry and Royle (2008) claimed unbiased estimates of species richness for occupancy models, Guillera‐Arroita et al. (2019) found that estimation bias can be substantial, in particular when 15% or more of the total richness has not been sampled. Inference on parameters representing changes in species richness might proceed differently from the estimat**i**on of species richness itself and patterns of bias and precision do not need to be the same for different parameters. A relevant case is found in the difference in estimation bias between estimates of population size and of individual survival between two time points (Cormack, 1972). Survival is a parameter determining changes in population size and is generally much less biased than population size estimators. Hillebrand et al. (2018) rightfully state that richness change in itself should be decomposed into trends in immigration and local extinction. Estimates of these rates might additionally be less biased than of species richness itself.

The analogy between estimating population size and species richness goes deeper. Estimation methods of the capture–recapture framework for individuals can be applied to assemblages of aggregated species encounter histories (MacKenzie et al., 2006; Nichols, Boulinier, Hines, Pollock, & Sauer, 1998; Nichols & Pollock, 1983). Each is a sequence of zeroes and ones indicating for which years at least one record is present for that species in the dataset. Williams, Nichols, and Conroy (2002) noted that the modeling of such species encounter histories when assuming open communities with colonization and extinction is similar to individual capture–recapture histories in the presence of temporary emigration. However, the dataset and the subsets analyzed here have no repeated within‐year occasions where the assemblage can be assumed to be closed, such that "temporary emigration," which here is a component of the colonization and extinction dynamics cannot be estimated because a robust design model cannot be fitted (Pollock, 1982). Temporary emigration, that is the probability that an individual is absent and cannot be sampled, can also be estimated for individual capture–recapture histories assuming an unobservable state (Kendall & Nichols, 2002; Schaub, Gimenez, Schmidt, & Pradel, 2004). Such a model as constructed for capture histories of individuals cannot deal with individual species jointly present inside and outside of the Netherlands and we would have to use occupancy models. For parameter estimability, we would further have to assume that there are no time trends in temporary absence and survival (Schaub et al., 2004) such that the time‐dependent models of interest cannot be fitted. Therefore, encounter histories per species were analyzed using capture–mark–recapture analysis for open populations (Pradel, 1996) and trends in temporary absence were assessed otherwise as explained below. Models were fitted to species encounter histories across years using Mark software (White & Burnham, 1999) called via RMark in R (Laake, 2013). I parameterized using the “Pradrec” model, which estimates local survival and colonization, where I note that the first is a probability per species (in between zero and one) and the second the number of colonizing species relative to the number present at the beginning of a time interval (non‐negative). Local survival probabilities, species colonization, and detection probabilities were estimated as being constant, with a linear trend over time (in the linear predictor), with categorical effects per year (“time‐dependent”), or with regression models that have the total number of records, number of grid cells with records per year, and *σ* of the Poisson lognormal distribution of the taxon concerned as year‐specific covariates (“regression”). This assumes that changes in the grid cells sampled primarily affect detection probabilities and that survival and colonization apply to the entire Netherlands. Correlated explanatory variables were jointly included in the detection probability model because the aim was to obtain the best predictions for survival and recruitment, not to interpret parameter estimates of the detection model or do hypothesis testing on them. The total number of records per species was added as a species‐specific (individual) covariate in some models. The Pradel models fit parameters that express per‐year gains and losses in species richness and not richness itself. This is expected to reduce estimation bias (Cormack, 1972) but it is also known that capture heterogeneity needs to be addressed (Abadi, Botha, & Altwegg, 2013) to avoid biased estimates of local survival and colonization. For that reason, mixtures of species effects on detection probability were fitted, with two or three components (“PradelRecMix” in RMark, Pradel, Choquet, Lima, Merritt, & Crespin, 2009). Models were compared using AICc to select the minimum adequate model with lowest AICc.

If the probability that a species is temporarily absent remains constant over time, it will not affect estimates of species richness change, only detectability. The presence of time‐inhomogeneous temporary absence could affect time trends in species richness and was assessed as follows. If the probability of being temporarily absent in year *t* is *p*_e_(*t*) and the probability of detection when present *p*_d_(*t*), then the probability of detection *p*(*t*) in the time‐dependent Pradel model should equal the product of *p*_d_(*t*) and (1 − *p*_e_(*t*)). Assuming that our regression model for detection probabilities with variables characterizing sampling effort and method fits in fact *p*_d_(*t*) multiplied by a constant, we can investigate the presence of elevated temporary absence in some time intervals by checking time patterns in the difference between detection probabilities predicted by the regression and by the fully time‐dependent models. Large positive differences in a sequence of consecutive years can indicate temporary absence affecting species richness trends.

Parameter estimates of local survival and colonization were used to calculate the change in species number in 2018 relative to 1945. The growth rate *λ_t_* of species richness in between year *t* and* t* + 1 is the sum of the survival probability *s_t_* and colonization *f_t_*. The predicted relative change in richness in year $\hat{t}+\tau$ relative to reference year $\hat{t}$ is equal to,

(1) $$\prod_{t=0}^{\tau -1}\left(s_{\hat{t}+t}+f_{\hat{t}+t}\right)$$

Confidence intervals for these changes were calculated from the confidence interval boundaries of parameters in the minimum adequate models.

To assess estimation bias in the parameters of Pradel models, simulations were used. Appendix S5 and Figures S3 and S4 present results of simulations of local survival, colonization, temporary absence and sampling, which allowed another assessment of estimation bias and of the statistical power to detect decelerating declines and temporary absence concentrated in a certain period.

## 3 RESULTS

All 44,355 and 197,827 records from 1945 to 2018 were included in the analysis of *Bombus* and non‐*Bombus* genera, respectively. The analysis of the data subsets had (a) 13,309 and 145,457 records for the analysis of specimens in collections and (b) 7,087 and 61,576 records from grid cells sampled in each of three periods.

### 3.1 Generalized nonlinear models

**Figure 2:**
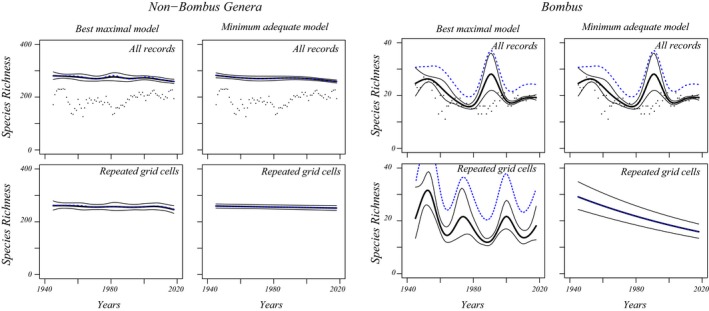
Time patterns of species richness obtained from generalized nonlinear modeling (gnlr). Model predictions and 95% confidence bands of the predicted values are drawn as full lines (confidence band limits: thin, estimates: thick). Per taxon, the left column shows predictions of maximum models with lowest AICc, the right column minimum adequate models. Blue dotted lines indicate predictions from a bootstrap‐bias‐corrected model and raw data points are added for comparison (black). Top row: models fitted to all data. Non‐*Bombus* genera. AICc non‐*Bombus* maximal model (*a*(*t*) 8 *df* spline of year, *b*(*t*) 8 *df* spline of *σ*): 419.9; AICc non‐*Bombus* adequate model (*a*(*t*) 3 *df* spline of year, *b*(*t*) 3 *df* spline of *σ*): 393.5. All data *Bombus*. AICc *Bombus* maximal model (*a*(*t*) 8 *df* spline of year, *b*(*t*) 8 *df* spline of year): 301.2; AICc *Bombus* adequate model (*a*(*t*) 8 *df* spline of year, *b*(*t*) 8 *df* spline of year): 301.2. Second row: data from 100 km^2^ grid cells visited in three periods. Left: non‐*Bombus* genera. AICc non‐*Bombus* maximal model (*a*(*t*) 8 *df* spline of year, *b*(*t*) 8 *df* spline of *σ*): 390.9; AICc non‐*Bombus* adequate model (*a*(*t*) 8 *df* spline of year, *b*(*t*) 1 *df* spline of *σ*): 373.4. Right: *Bombus*. AICc *Bombus* maximal model (*a*(*t*) 8 *df* spline of year, *b*(*t*) 8 *df* spline of year): 350.0; AICc *Bombus* adequate model (*a*(*t*) 1 *df* spline of year, *b*(*t*) 3 *df* spline of *σ*): 327.4

Prediction intervals of the best maximal models and the models with the most favorable AICc (adequate model) are shown in Figure 2 for the full data and the second subset, that is records from grid cells sampled in three predefined periods (results on collection specimens are available in Figure S1, Table S1).

The overall impression from Figure 2 is that there are moderate declines of species richness for the non‐*Bombus* genera. For *Bombus*, the analysis of the complete dataset produces a complex pattern in species richness over time. The complexity disappears when the data are restricted to grid cells that have been sampled in three periods and when we consider the minimum adequate model. Comparison with the analyses on the other grid cells that were not sampled in three periods (Appendix S4, Figure S2) reveals that the complex pattern might originate from this data subset and thus from data heterogeneity. For the minimum adequate model on collection specimens only, a straight line remaining within the prediction intervals can be drawn from the start to the end of the study period (Figure S1).

The bootstrap‐bias assessment shows that the predictions of bias‐corrected models remain within the original confidence intervals for the non‐*Bombus* genera but not for *Bombus* (Figure 2). There, the bootstrap predicts substantial bias. A first explanation is that this is due to the fact that I did not add rare species in the bootstrap procedure (Chao et al., 2014) and thus never sampled more species than in the data for that year. The bootstrap bias disappears in the minimum adequate model for the grid cells that were repeatedly sampled (Figure 2). This suggests that bias might be larger when complex models overfit the bumblebee data. With overfitting, sampling variation is interpreted as part of the nonlinear regression. I therefore conclude that sampling heterogeneity and overfitting lead to spurious patterns and that we should infer a constant decline for bumblebees.

For non‐*Bombus*, species richness in 1945 is estimated from the maximal model as 281 (95% confidence interval [267, 296]), in 2018 as 259 [251, 268]. For *Bombus*, the estimate for 1946 is 25 [20, 30], by 2018 richness is 19 species [18, 20]. Note that the estimated relative loss of richness is larger for *Bombus* than the other bee genera but when relative losses are calculated from confidence intervals limits, they overlap and both touch zero loss.

### 3.2 Capture–recapture analysis

**Figure 3:**
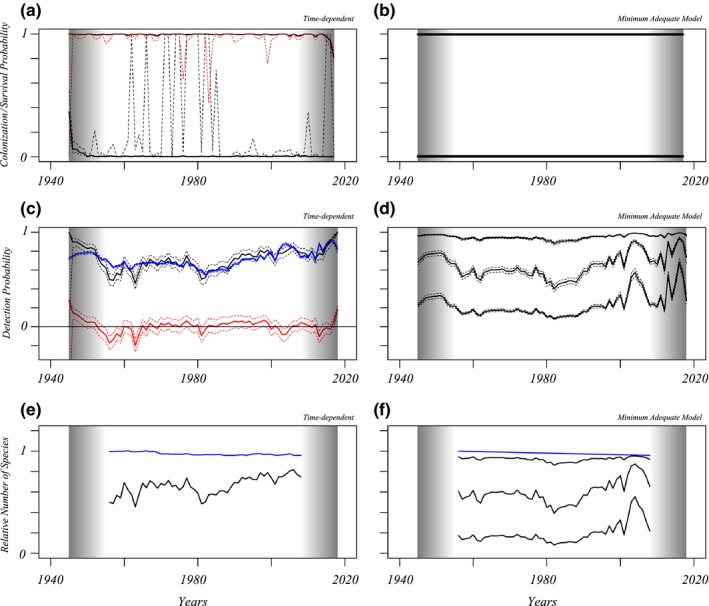
Results obtained from fitting Pradel models to encounter histories for species from non‐*Bombus* wild bee genera. Left column: Parameter estimates and predictions of the fully time‐dependent model without fitting mixtures; right column: the adequate model with a mixture for detection probability (Table 1). Top row: Local survival (upper full curve) and colonization probabilities (lower full curve) per year, which are close to values one and zero, respectively. Confidence intervals are indicated as dotted lines, in red for colonization probabilities. Middle row: Detection probabilities. In the left panel the predictions of a model with categorical time effects (black) and a model with logit regressions of the three year‐specific explanatory variables (blue) are plotted with 95% confidence intervals. The differences between both are plotted in red and can be informative on the occurrence of temporary emigration. Right panel: For the minimum adequate model, detection probabilities vary between species, and the curve plotted is for each of the three components in the species mixture. Bottom row: Trends in relative species richness over years, where 1955 is used as a reference and given value 1 (blue: species richness, black: predicted relative number of species sampled). In the bottom right panel, the predicted relative number of species sampled is plotted for each mixture component. Gray bands: biased parameter estimates for survival and colonization are expected for these years on the basis of similar analyses over shortened time intervals

**Figure 4:**
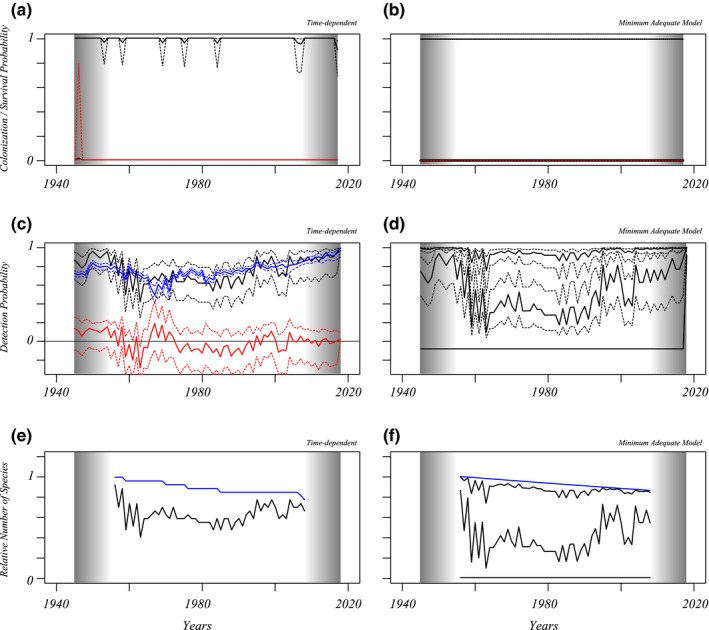
Results obtained from Pradel models fitted to species in the *Bombus* bumblebee genus. Order and content of all panels are as for Figure 3. For *Bombus*, detection probability is a mixture of three components, of which one group of species has negligible detectability. Additional information on the adequate model can be found in Table 1

Figures three and four show the results of fitting time‐dependent (left, no mixtures) or minimum adequate Pradel models (right) to the data. Reducing the datasets to shorter intervals and refitting models revealed that the estimated survival and colonization patterns near the start and the end of encounter histories are biased in models with categorical time effects. In both groups of species, models with detection probabilities that are mixtures of several components without year effects on local survival nor colonization were preferred (Table 1, Figures 3 and 4). The time‐dependent models show negligible colonization and some years with reduced survival. Table 1 gives AICc values of the adequate models and a set of other models that are useful for comparison. In these tables, “*t*” indicates time categorical effects fitted, “*T*” a linear effect in the linear predictor, which was linked to the data using log (colonization) or logit (local survival) link functions. The number of mixture components in the detection model is given. In the last two columns of the tables, estimates of the linear effect of *T* are given when fitted. At least one estimate for local survival or colonization needs to be significantly positive for a deceleration in the decline of species richness to be possible. This does not occur in the analyses of the different data subsets either (Table S2). However, this is in agreement with simulation results (Appendix S5, Figure S3) that indicate that there is a bias toward estimating negative trends in local survival. However, the simulations also indicate that survival intercept parameters are estimated relatively well, such that we can use these to estimate species richness change while our minimum adequate models are not including any time effects on survival or colonization. When time‐dependent and regression models are compared (no mixtures, Figures 3 and 4, second rows left), there are a limited number of years where detection probabilities differ significantly between these models, once for bumblebees and repeatedly for the non‐*Bombus* genera. Simulations (Appendix S6) show that this approach can indeed detect temporary absence of species, with limited power. The years in between 1955 and 1965 could be years where a fraction of non‐*Bombus* species temporarily disappeared. However, the period is brief and early in the time series such that the species are expected to have been present again since 1965. Based on parameter estimates of the models with lowest AICc, in 2018 the species richnesses of non‐*Bombus* wild bees and of *Bombus* bumblebees have decreased 6% (confidence interval [−6, 16%] and 19% ([10, 34], colonization fixed at zero), respectively, since 1946. Simulations (Figure S4) indicate that these values might be estimating the real decreases relatively well.

**Table 1:** A selection of Pradel models fitted to the data. The minimum adequate model is given on the first line per taxon. The first three columns list the covariates in the models for local survival, colonization and detection, the last two columns confidence intervals for the time trends in survival and colonization, when estimated. *T* refers to a linear effect of year, *t* to categorical effects of year, “1” indicates a model without explanatory variables. The numbers of components in the mixture distributions for detection probabilities are given. Abbreviations for the year‐dependent variables are *σ* for the heterogeneity parameter of the Poisson‐log normal distribution, *N*_rec_ is the total number of records per year, *N*_grid_ the number of square kilometer grid cells sampled

Survival	Colonization	Detection	Deviance	Npar	AICc	Estimated change in survival	Estimated change in colonization
(a) Non‐*Bombus* Bees—All data 1945–2018							
*1*	*1*	*N*_gr_, *N*_rec_, *σ*, 3 components	13,600	9	17,127	NA	NA
*T*	*T*	*t*, 2 components	14,084	80	17,753	[−0.026, −0.001]	[−0.028, −0.004]
1	1	*t*, 2 components	14,447	78	18,113	NA	NA
*T*	*T*	*N*_gr_, *N*_rec_, *σ*, 2 components	14,593	10	18,122	[−0.027, −0.002]	[−0.022, −0.009]
*t*	*t*	*T*	20,753	220	24,709	NA	NA
(b) *Bombus* Bumblebees—All data 1945–2018							
1	1	*t*, 3 components	1,203	79	1,552	NA	NA
*T*	*T*	*t*, 3 components	1,202	81	1,555	[−0.052, −0.013]	[−183, 182]
*1*	1	*N*_gr_, *N*_rec_, *σ*, 2 components	1,369	8	1,566	NA	NA
*T*	*T*	*N*_gr_, *N*_rec_, *σ*, 2 components	1,369	10	1,570	[−0.037, 0.034]	[−62, 62]
*t*	*t*	*T*	1,684	220	2,390	NA	NA

## 4 DISCUSSION

To investigate trends in species richness, a dataset with records and observations of *Bombus* and non‐*Bombus* wild bee species in the Netherlands spanning 73 years was analyzed using two approaches. In the analysis of such an opportunistic dataset, emerging patterns can be caused by sampling heterogeneity across years, while there is insufficient data to model all potential sources of sampling heterogeneity adequately within a single elaborate model. Therefore, I proposed that a constant decline in species richness should only be rejected when we can also do so when records are removed from spatial locations that only contributed data in a restricted time interval and when records are removed that do not correspond to samples in natural history collections. This was the main source of records before 2000, but now contributes a minor fraction of the data. Neither of the approaches then rejected the hypothesis of a constant decline or no decline at all in favor of a decelerating decline. In fact, the species richness decline is small and maybe absent for non‐*Bombus* bees. For bumblebees, if we take advantage of the larger precision of the capture–recapture analysis, a moderate and significant decline of 19% is inferred. Among the generalized nonlinear models, there was one data subset for which the preferred model had a constant decline in species richness for both bumblebees and other wild bee species, while preferred models for the other subset and the full dataset had smooth regressions of species richness with at least three parameters. Therefore we can conclude that the approach is capable of selecting a nonlinear model, but it did not do so consistently. Several estimation issues were encountered. Generalized nonlinear modeling showed potential estimation bias for bumblebees, which might result from the lack of adding rare species in the bootstrap, or from fitting too complex models to the data. Simulations indicated that the modeling of species richness changes via encounter histories suffers from estimation bias of parameters representing time trends, making it difficult to reliably detect decelerating declines when using that method alone.

### 4.1 Wild bee species richness trends

The fraction of species richness lost since 1945 or the species richnesses at the end of the study period could be predicted using either approach. From the capture–recapture analysis, the conclusion is that species richness has declined since 1945 for the *Bombus* genus. For the non‐*Bombus* genera small declines are estimated with confidence intervals including zero. The decrease estimated for the *Bombus* genus is comparable to a study using rarefied richness on Swedish data (Bommarco, Lundin, Smith, & Rundlöf, 2011). On the other hand, the estimated overall decrease for the other genera seems smaller than in an analysis of bee species richness in the UK with relative decreases between 10% and 30% in most study sites, when comparing periods separated by 33 years (Senapathi et al., 2015). However that study used the same methods as Carvalheiro et al. (2013) and no confidence intervals for the changes were given. Again for the UK, Ollerton, Erenler, Edwards, and Crockett (2014) analyzed bee and flower‐visiting wasp species richness and noted extinctions before 1960, which they attributed to agricultural intensification. These authors noted that their results for the most recent decades contradict Carvalheiro et al. (2013), as extinctions might be increasing again. Van Strien et al. (2016) observed a modest recovery of biodiversity in the Netherlands, measured as the living planet index, from 1990 to 2014. It is concentrated, however, in the freshwater habitats and with diversity decreases in the habitats where wild bees typically occur.

The stronger decrease suggested for *Bombus* bumblebees is surprising, given that bumblebee densities and presences are assumed not to be determined by very local landscape characteristic and can benefit from urbanization (Carré et al., 2009; Kennedy et al., 2013; Senapathi, Goddard, Kunin, & Baldock, 2016). However, bumblebees might show an elevated susceptibility to rapid climate change (Kerr et al., 2015).

Combining the results on Dutch wild bees and bumblebees with the reassessment of Van Dooren (2016), the conclusion must be that there are no decelerations in the declines of species richness in pollinators in northwest Europe. There is thus no reason to be satisfied with current biodiversity conservation efforts, as there is no or insufficient evidence that they have been effective. It has been stated elsewhere that the most recent EU CAP Agricultural Reform fails on managing biodiversity adequately (Pe'er et al., 2014). While it has been shown that visitation to crop flowers increases with the number of species per field (Garibaldi et al., 2013), species richness might be not the key quantity predicting crop pollination services to agriculture, as common species provide most of these (Winfree, Fox, Williams, Reilly, & Cariveau, 2015). Kleijn et al. (2015) therefore state that conservation and immediate utility goals for agriculture might not align. However, it might just be a matter of time before they do when environments continue to change. Inferring arrested biodiversity declines as was done by Carvalheiro et al. (2013) appears dangerous and I prefer to consider the small estimated loss for non‐*Bombus* bees as meaningful and potentially indicating an actual trend.

### 4.2 Methodological developments

Risks of species richness loss seem taxon‐specific (*Bombus* vs. non‐*Bombus*), and thus call for trend analysis in smaller taxonomic groups in general. However that requires sufficient data for each of them. Boyd (2013) has proposed that audited standards robust to variations in assessor competence should be available and used in biodiversity research and data collection. For historical data, it is too late for that. Retrospective sampling standardization is impossible. With data of the type analyzed here, we therefore need to resort to detailed statistical modeling, data inspection and model checking, with conservative inference to avoid new erroneous conclusions. The situation could have been as bad as O'Hara (2005) suggested, namely that we often rather analyze properties of estimators than richness patterns themselves. This is confirmed for the estimation of time trends in local survival using Pradel models, where estimation bias leads to wrong inference and masks differences between datasets. The generalized nonlinear modeling does not need to assume a constant presence or absence probability, and the manner in which the sampling process is modeled comes with limited assumptions. However, the method needs manual work, as model fitting is tedious. Convergence to a global maximum likelihood solution is not guaranteed and results need to be inspected with care and can suffer from overfitting.

The results of each analysis presented here urge further methodological developments. Chao et al. (2014) proposed a bootstrap method to estimate variances of extrapolated richness, where the fraction of rare missing species is first estimated and then added to an imputed dataset from which resampling occurs. I have used bootstrap methods differently, to estimate magnitudes of estimation bias in the different types of analysis but without imputing rare species. For assessments of estimation of bias, independent simulations seemed more useful than the data bootstrap.

Issues with models for encounter histories are probably alleviated when robust design models (Pollock, 1982) can be fitted to the data. If records had been collected throughout the study period independently by observations and via specimens deposited in collections, then these two sampling methods could have been used to fit a robust design. Heterogeneity in species detection probabilities can be expected in all methods addressing species richness (e.g., Boulinier, Nichols, Sauer, Hines, & Pollock, 1998) and it was incorporated into the capture–recapture models by means of mixtures. Note that models for open populations were used in the analysis of species encounter histories. Models exist to estimate species richness in open occupancy models (Nichols et al., 1998; Yamaura et al., 2011), but these often depend on the assumption of individual random sampling which is untenable for the opportunistic data analyzed here. Statistical modeling of how collectors vary in their sampling efforts over time clearly deserves more study and wider application, given that more and more data of the kind analyzed here are mined and as people are encouraged to collect citizen‐science data (Potts, Imperatriz‐Fonseca, Ngo, Biesmeijer, et al., 2016). Ideally, these developments should result in methods where local or landscape‐specific diversity trends and spatio‐temporal patterns in sampling effort can be studied jointly. Also studies on abundance trends in different taxa (e.g., Inger et al., 2015) could benefit from multimodel inference and assessments of bias‐variance tradeoffs and sampling heterogeneity.

Isaac and Pocock (2015) propose that an effort should be made to model observer responses, suggesting the application of data collected from mobile phones to assess different kinds of sampling bias. This seems to call for an effort to model recording syndromes and to integrate them into the development of statistical methods for uncontrolled data collection.

A very different avenue of research which could be rewarding might be the development of Focused Information Criteria (FIC; Claeskens & Hjort, 2003) for the estimation of species richness trends. Such information criteria have a focus (hence the F), a statistic we are most interested in, and models are preferred that are expected to produce the most precise predictions of that statistic. We would thus require a FIC for the selection of models that predict species richness trends best.

Meanwhile, how can we decide which approach is reliable enough for inference of trends? Here I resorted to comparisons of consistency in the results from different approaches and subsets. Additional simulations with similarities to the dataset were essential and revealed critical estimation bias. Therefore, ranking estimates from different approaches based on consistency across data subsets should never occur without accounting for other criteria. For opportunistic data, there seems to be currently no safe alternative other than a pluralistic but conservative modeling approach assisted by simulations which provide some guidance.

To conclude, I want to point out a potential example of how nonrandom data collection might be generated. Note that a recent Dutch reference work on wild bees (Peeters et al., 2012) explicitly pointed to recent records of the wild bee species *Andrena coitana* in Germany and the habitat type in the Netherlands where the species might be seen again after a long period without records. Recently, the rediscovery of *A. coitana* was reported (Nieuwenhuijsen, 2016). We cannot exclude the possibility that a new reference work or other public exposure motivates an increased effort to collect particular species. This can thwart any effort to achieve random sampling, so essential to most of our inference methods, by replacing it with recording syndromes (Isaac & Pocock, 2015).

## CONFLICT OF INTEREST

None declared.

## AUTHOR CONTRIBUTION

TJMVD designed the study, carried out the analysis and wrote the manuscript.

## Open Research Badges

This article has earned an https://openscience.com for making publicly available the components of the research methodology needed to reproduce the reported procedure and analysis. All materials are available at [https://doi.org/10.5061/dryad.34tmpg4fm].

## Data Availability

The raw data used are available upon request from http://www.eis-nederland.nl/. Scripts with the data analysis are available on Dryad at https://doi.org/10.5061/dryad.34tmpg4fm.
