# Supplementary material for: Assessing species richness trends: Declines of bees and bumblebees in the Netherlands since 1945
Source: Ecol Evol. 2019 Nov 7;9(23):13056–68. doi: 10.1002/ece3.5717 (PMC6912890; doi:10.1002/ece3.5717)
Supplement: Supplementary file 1 [file ECE3-9-13056-s001.docx]

Appendices with "Assessing species richness trends: declines of bees and bumblebees in the Netherlands since 1945".

Tom JM Van Dooren 20 July 2019.

Complete and self-explanatory R scripts of the analysis and data frames with variables are available from the author as a compressed .zip file or can be found on Dryad (upon acceptance of the manuscript for publication). While the main text discusses two approaches for modelling species richness change, here I present four. The first is mainly there to illustrate that counts are the natural kind of random variables to represent species richness. The second was an attempt to integrate estimates of species richness obtained using independent software into a regression framework which respects that the variances of the estimates are heteroscedastic.

Appendix S1. Modelling species number accounting for sample coverage.

Chao and Jost (2012) have proposed to compare species numbers at fixed sample coverage. Using species number data, one can construct a simple linear model that includes years and sample coverage as explanatory variables. It can predict species richness at a sample coverage equal to one.

For species numbers "Sp", years "Years", sample coverage "Coverage", and assuming a simple quadratic model for the year effects the model is

model<-glm(Sp~Years+Years^2+I(1-Coverage),family=poisson)

# predicted richnesses at sample coverage = 1

predicted_richness<-predict(model,newdata=list(coverage=rep(1,69)),type="response",se.fit=T)

I did not pursue this generalized linear modelling approach further, for example by modelling standard deviations of species numbers as in Appendix S2 and S3. It is unlikely that the data in this paper were obtained using random sampling. Estimates of sampling coverage might therefore be inadequate and their precision difficult to assess. We can expect attenuation of the coverage slope, because coverage is not exactly known. There are methods to take that into account, which do assume that coverage estimates are not biased. The correction for coverage is linear, therefore the range of coverage values in the data should be limited to make this approximation valid. This method might perform well for randomly sampled data and different subsets per year with slightly different coverage values so that there is coverage variation independent of years.

Appendix S2. Smooth regressions of species richness estimates.

In this second type of analysis, I used species richness estimates in smooth regression models with several covariates. Covariates can represent factors that might explain bias in estimation, or a change in the spatial extent of the assemblage where richness is assessed. These are by necessity changing over time, just as the year covariate which is fitted to estimate time trends. This approach thus does not have a clear part of the model representing the observation process, except for the fact that some covariates related to it are used in smooth regressions. As estimates of species richness, a non-parametric estimator can be used, a specific parametric estimator or the "best" estimator selected among five different parametric models using a detailed heuristic (Bunge et al. 2012). Program CatchAll (Bunge et al. 2012) was used to obtain these estimates of species richness per year, with an estimated standard deviation. Time patterns in log annual species richness estimates were then modelled using smooth functions of the following explanatory variables: the year where the samples or records were from, the number of square kilometre grid cells with records per year, and the estimated variance parameter *σ* of the Poisson lognormal distribution fitted to numbers of records per species per year for the taxonomic group analysed. These smooth regressions of log-transformed species richness were fitted using the gamlss() function for R (Stasinopoulos & Rigby 2007). I modelled heterogeneous errors in log species richness. I fitted as maximal models cubic splines of 9 d.f. for the year variable, and of 4 d.f. for the other explanatory variables (note that d.f. in gamlss() output are given as on top of the linear model). First the model was simplified by reducing the d.f. of the cubic spline for the year variable, subsequently by doing that for the other variables. Models were compared using AICc as for the models in the main text, until a minimum adequate model with lowest AICc was identified.

A maximal model fitted in R using the gamlss() function can be written at follows:

gamBPnb8<-gamlss(formula=log(pred)~cs(year,df=8)+cs(poilogsigma)+cs(Ngrids),

sigma.formula=~offset(exp(predse/pred)))

Here, “pred” is the predicted species richness, “year” is the calendar year, “poilogsigma” is the sigma parameter, “Ngrids” the number of square kilometre grid cells with samples in each year and “predse” is the estimated standard error of the predicted species richness.

I decided not to present this method in the main text as it has limited separate modelling of the sampling process. The regression includes several time-dependent variables which can be highly correlated, complicating hypothesis testing. There can be many heuristic inference steps in between data collection and the regressions (when selecting the "best" estimator), for which the smooth regression modelling cannot account.

Appendix S3. Generalized non-linear regression.

This approach and some of its characteristics are also presented in the main text. None of the estimation methods proposed by Raaijmakers (1987) nor the non-linear Least Squares used by O'Hara (2005) were applied. Here I paste the code for one of the most elaborate models fitted using gnlr (Lindsey 1997), including the initial values for all parameters used in the ML optimization. In the short script below, “Sp” is the number of species, “spl8” is a natural cubic spline of eight d.f. of the year variable, ”rec” is the number of records per year. “SD” is the predicted unconditional standard deviation of the number of species at the actual number of records, calculated following Colwell et al. (2012).

gnlrmodel<-gnlr(sp,mu=~exp(a+a1*spl8[,1]+a2*spl8[,2]+a3*spl8[,3]+a4*spl8[,4]+a5*spl8[,5]+a6*spl8[,6]+a7*spl8[,7]+a8*spl8[,8])*rec/(rec+exp(b+b1*spl8[,1]+b2*spl8[,2]+b3*spl8[,3]+b4*spl8,4]+b5*spl8[,5]+b6*spl8[,6]+b7*spl8[,7]+b8*spl8[,8])),

pmu=c(1,0,0,0,0,0,0,0,0,1,0,0,0,0,0,0,0,0),

shape=~log(exp(v0)+SD^2),pshape=list(v0=-2))

For this model, the predicted species richnesses across years are given by

exp(spl8bb%*% gnlrmodel$coef[2:9]+ gnlrmodel $coef[1])

Figure S1. **Results when modelling different subsets** **separately**. Results of generalized non-linear models, containing models also presented in Fig. 2. Analysis of the full datasets in the top row. Analyses of non-*Bombus* and *Bombus* records for which specimens were deposited in museum collections (middle row). Models using data from grid cells sampled in each of the three time periods as defined in the main text are shown in the bottom row. Estimates of species richness (gnlr) and confidence bands of selected minimum adequate models are given in Table S1 below.


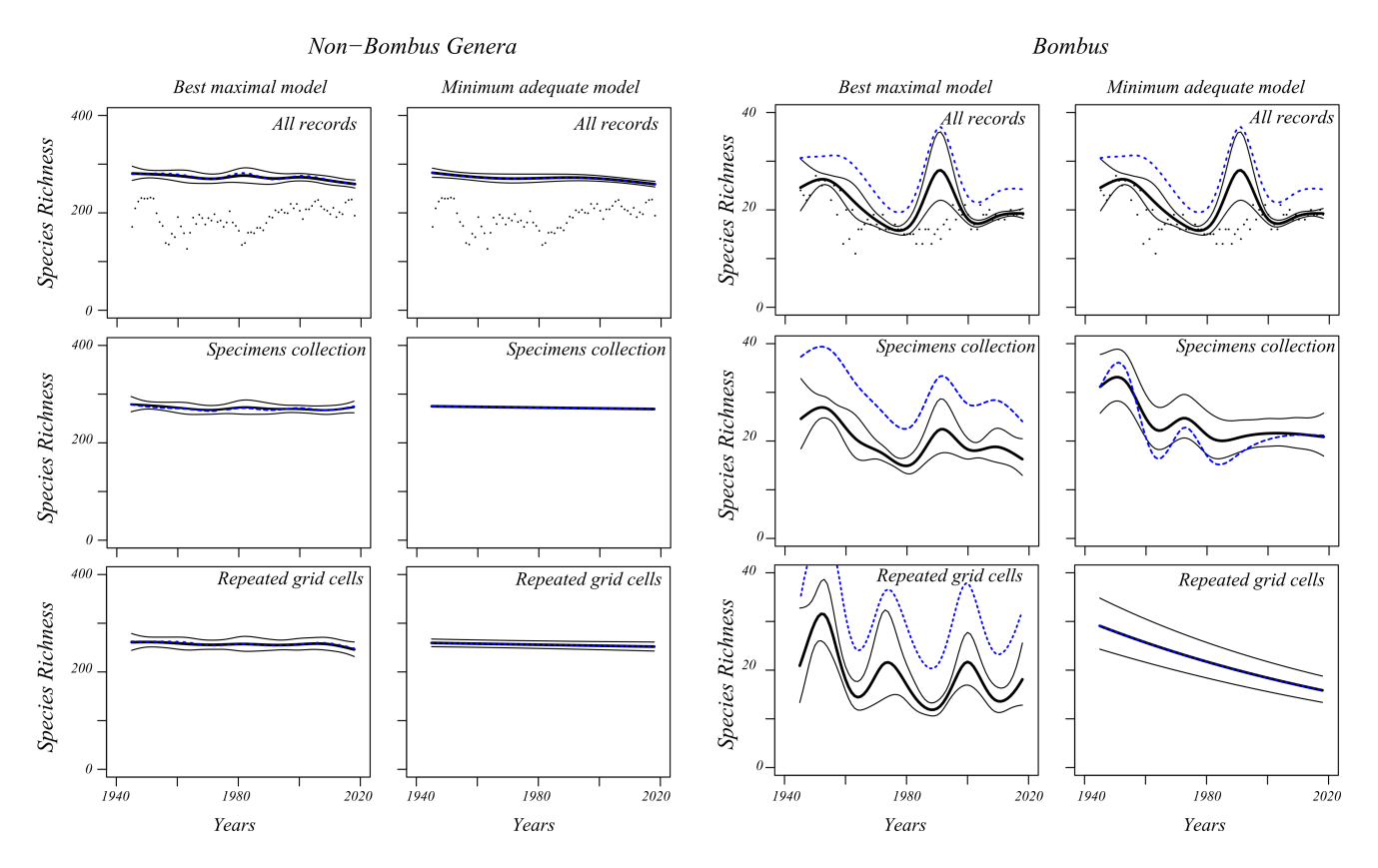


Table S1. Adequate models obtained for the gnlr analyses of all data and the two data subsets. The explanatory variables and the degrees of freedom of the splines used to model *a*() and *b*() are given.

| non-*Bombus* | All | Collection | Grids |
| --- | --- | --- | --- |
| Gnlr | *a*(year, d.f. = 3)  *b*(*σ*, d.f. = 3) | *a*(year, d.f. = 1)  *b*(*σ*, d.f. = 1) | *a*(year, d.f. = 8)  *b*(*σ*, d.f. = 1) |
| *Bombus* |  |  |  |
| Gnlr | *a*(year, d.f. = 8)  *b*(year, d.f. = 8) | *a*(year, d.f. = 8)  *b*(*σ*, d.f. = 1) | *a*(year, d.f. = 1)  *b*(*σ*, d.f. = 3) |

Table S2. Pradel models fitted to all data and to the data subsets. Minimum adequate models are given among the models with estimates of time trends of colonization and survival. Abbreviations of explanatory variables are as in Table 1, In addition, *N*_sp_ is the number of records per species used as a species (individual) covariate.

| Table S2a. Non-*Bombus* Bees – Time trends for each subset analysis | | | | | |
| --- | --- | --- | --- | --- | --- |
| Survival | Colonization | Capture | Subset | Estimated change in survival | Estimated change in colonization |
| *T* | *T* | *t,* 2 components | All data 1945-2018 | [-0.026, -0.001] | [-0.028, -0.004] |
| *T* | *T* | *t*,  *N_sp_* | Collection | [-0.024, 0.004] | [-0.040, -0.010] |
| *T* | *T* | *N_gr_* , *N_­rec_*, *σ*, *N_sp_* | Grids | [-0.036, -0.009] | [-0.023, 0.010] |
| Table S2b. *Bombus* Bumblebees – Time trends for each subset analysis | | | | | |
| Survival | Colonization | Capture | Subset | Estimated change in survival | Estimated change in colonization |
| *T* | *T* | *t*, 3 components | All data 1945-2013 | [-0.052, -0.013] | [-183, 182] |
| *T* | *T* | *N_gr_* , *N_­rec_*, *σ*, *N_sp_* | Collection | [-0.050, 0.015] | [-10.0, 40.3] |
| *T* | *T* | *N_gr_* , *N_­rec_*, *σ*, 3 components | Grids | [-0.048, 0.056] | [-67.7, 65.4] |

Appendix S4. Generalized linear models fitted to grid cells that were not repeatedly sampled in different time periods.

Here I report on the analysis of the dataset consisting of records from grids that were not in the subset presented in Figure two (second row). These data consist of 136251 records of non-*Bombus* bees, and 37268 records of *Bombus* bumblebees.

Among the maximal models for the non-*Bombus* genera, the AICc for the model (*a*(t) 8 d.f. spline of year, *b*(t) 8 d.f. spline of *σ*) was lowest, AICc = 431.7. The minimum adequate model was (*a*(t) 6 d.f. spline of year, *b*(t) 3 d.f. spline of *σ*) with AICc equal to 411.9.

Among the maximal models for the *Bombus* bumblebees, the AICc for the model (*a*(t) 8 d.f. spline of year, *b*(t) 8 d.f. spline of *σ*) was lowest, AICc = 302.9. The minimum adequate model was (*a*(t) 8 d.f. spline of year, *b*(t) 3 d.f. spline of *σ*) with AICc equal to 291.0.

Figure S2. **Time patterns of species richness obtained from generalized non-linear modelling** (gnlr) on the subset of the data restricted to grid cells that were not repeatedly sampled as explained in the main text. Model predictions and 95 % confidence bands of the predicted values are drawn as full lines. Left column: best maximal models. Right column: minimum adequate models. Maximal and minimum adequate models and their AICc are detailed in the text above (Appendix S4).


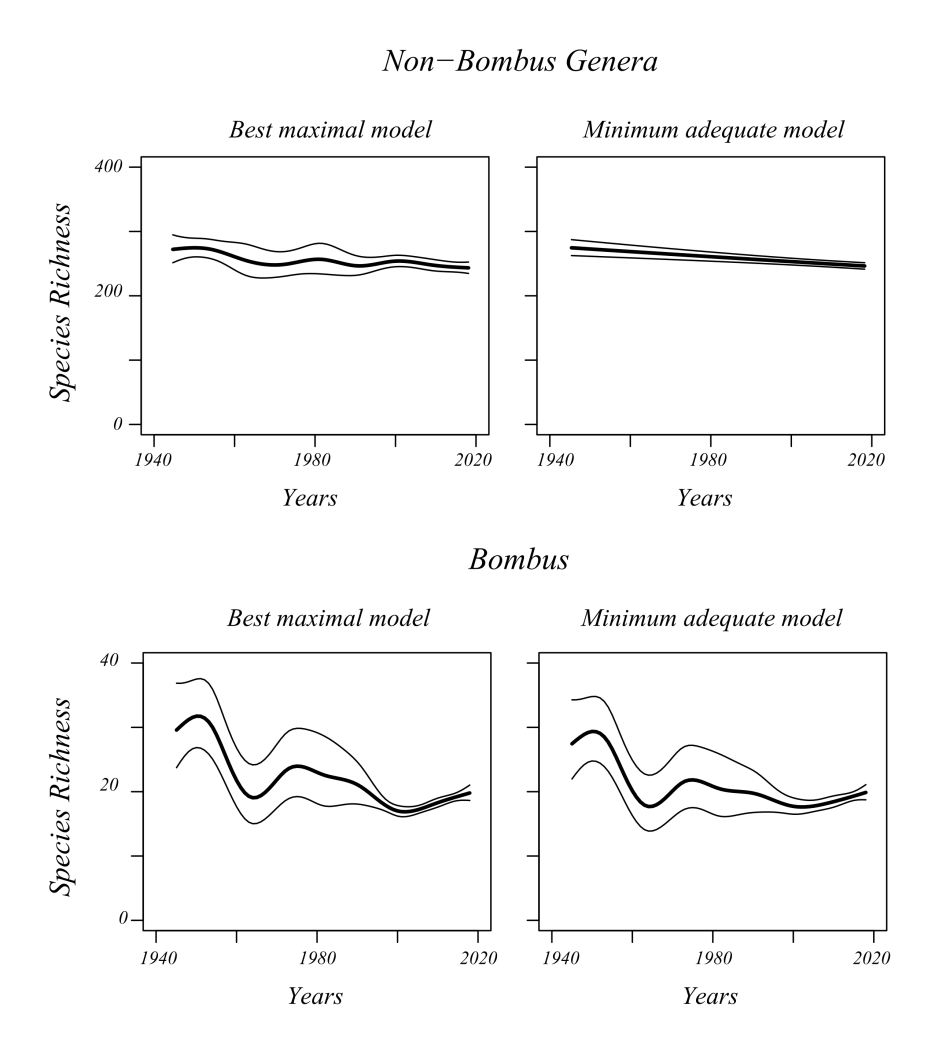


Appendix S5. Simulations of species change and estimation bias.

Changes in bee species richness were simulated using the relative numbers of individual records per species in the data as proxy for the initial relative abundances. All simulations started with 325 non-*Bombus* or 28 *Bombus* species. Only species local survival was modelled, and no colonization. Simulation code is available as a text file. Simulated assemblage time series were randomly sampled to match the numbers of records per year in the actual data and to produce time series of simulated records. Different effects on local survival were considered: 1) different time trends in species survival probabilities, 2) effects of relative abundance on the probability that a species survives, 3) different average survival probabilities (intercepts) at the start of the time series.

To simulate survival, we drew per year and species a random binary variable with average probability of success (local survival) of

$p=invlogit(\beta_{0}+\beta_{y}year+\beta_{ra}x_{ra})$ (S1)

with *year* for the year regression effect and with *x_ra_* for the relative abundance of the species in that year. Parameter values for *β*_0_ were (4, 5, 6, 7), for *β*_1_ (-0.02, 0, 0.02, 0.05), for *β*_2_ (0, 0.0005, 0.0010). Per combination of parameter values, three replicates were simulated and simulations were repeated for *Bombus* and *non-Bombus* genera separately, with similar results. To each simulated dataset, different Pradel models were fitted. All estimated a constant colonization probability. Local survival was either constant, with a regression of the year effect, with an effect of the total number of records per species, with additive effects of both. Detection probabilities were fitted with a regression of the year effect, with effects of *σ* and the number of observations, with the last two effects and the total number of records. A model with constant survival and colonization and a regression for detection probability with *σ* and the number of observations was usually preferred. Only results for the non-*Bombus* genera are discussed and shown below.

The main results for the models that do estimate year effects are the following. First, the intercept estimate for survival appeared relatively unbiased (Fig. S4, left). Second, year regression effects are underestimated across models, with stronger underestimation of positive trends in the probability to survive and stronger underestimation when the local survival probability per year is large (Fig. S3, middle). Third, immigration was overestimated. The intercept for colonization per year was estimated to be 0.0029 on average across all simulations (s.d. 0.0013), while we did not simulate colonization at all (Fig. S3, right). Fourth, when species richness change is estimated across the entire simulation period (50 years), including colonization in the calculation leads to underestimation of the decrease in richness across the period, setting recruitment to zero leads to rather unbiased estimation of the decrease (Fig. S4).

Fig S3. **Estimates of the intercepts for local survival (left), the time trends in local survival (middle) and colonization rates (right), across all simulations without effects of relative abundance on survival**. All estimates shown are from fitted models with year regression effects on survival and regression models of the explanatory variables for detection probabilities.. To visualize the pattern in the results, separate regression models were fitted to the estimates per simulated intercept for local survival *β*_0_, showing the underestimation in dependence on this simulated intercept. For reference, the true values set in the simulations are indicated by blue lines. In the middle panel, the point estimates for each regression have a different colour. It is shown as a dot to the right of the panel, next to the corresponding regression line.


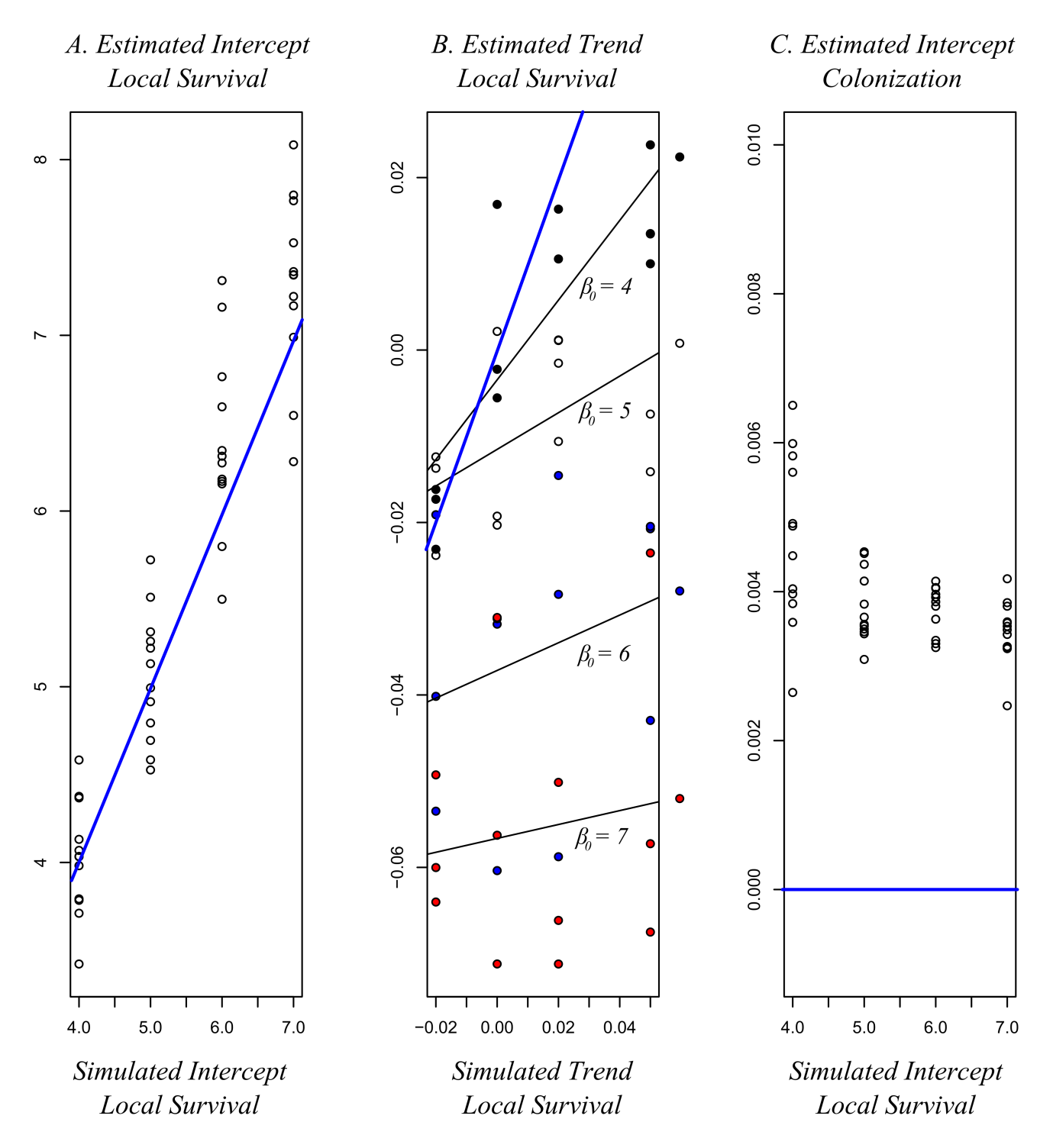


Fig S4. **Estimated species richness at the end of each simulation, in function of the actual remaining species richness of that simulation after fifty time steps**. A blue diagonal indicates where actual and predicted richnesses are equal. Red points are from calculations that include estimated local colonization effects. Black points estimate species richness on the basis of local survival only. Regression lines for the red and black points are added.


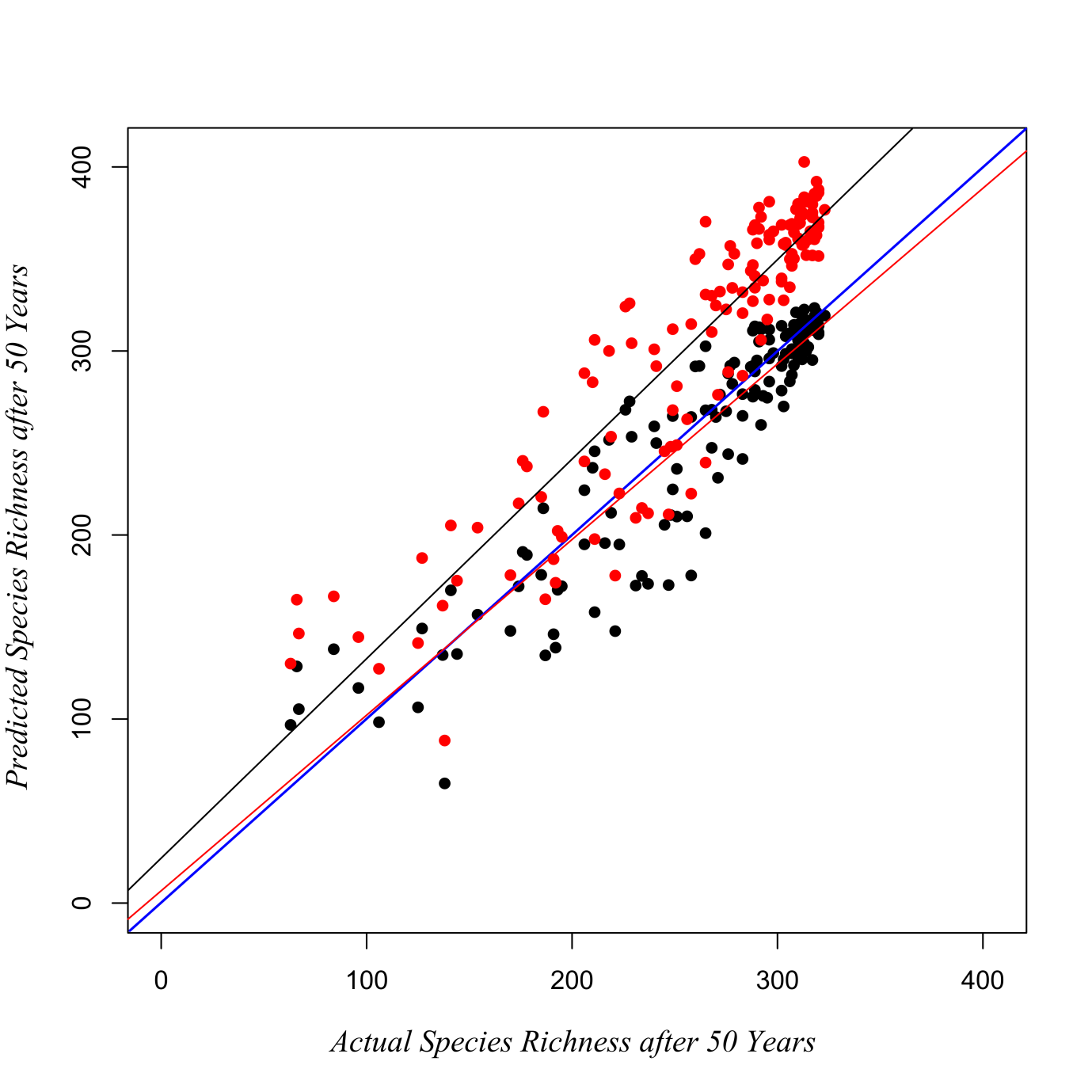


Appendix S6. Checking for the occurrence of temporary absence.

The main text states that the models for detection probability suggest a limited presence of temporary absence of species. Here I present further investigation of the issue.

It is implausible to assume that there would have been no temporary absence of individual species in this study at all. Temporary absence will affect estimation of species richness trends only if species have disappeared entirely from the study area for a substantial period, and have returned afterwards, and in such a way that the probability of temporary absence was larger for a fraction of the time period. I simulated such temporary absence of 10% of the species present between years 20 and 30 of a simulation running over fifty years (simulation code available as a text file). A model with time trends in local survival and constant colonization was fitted to the resulting data, and detection probability either with categorical effects of time or a regression of covariates as in the main text. The differences between detection probabilities predicted by the two models were calculated (Figure S5, non-*Bombus*) and limits of confidence intervals compared. For the *Bombus* genus, we could basically not detect temporary absence. For non-*Bombus* and between twenty and thirty years in the simulation, 19% of the confidence intervals of the predicted detection probabilities are non-overlapping for this effect size and in the direction we expect in the presence of temporary absence (Fig. S5, bottom). The difference between the lower limit of the prediction confidence interval of the regression model and the upper limit of the prediction interval of the time-dependent model is positive. In the other years, 1% of the confidence intervals are significantly different in this manner. These are mostly concentrated in a single year (Fig. S5).

Figure S5. **Results of simulations based on non-*Bombus* relative abundances where temporary absence occurred for 10% of the species between years 20 and 30**. Top panel: differences per year between predicted detection probabilities of the categorical time-dependent model and the regression model. A generalized additive model with an automatic smoother estimation and selection procedure (method "GCV-Cp"; Wood 2006) was fitted to the differences and is drawn as a blue line. Bottom panel: separation between confidence intervals of predicted detection probabilities in the regression model and the categorical time-dependent model. When this separation is positive, the detection probability is significantly larger for the regression model, hence the categorical time effect is significantly smaller. This can indicate temporary absence of a fraction of the species. Please note that positive separation values are concentrated between years twenty and thirty, where we simulated a temporary absence of 10% of the species.


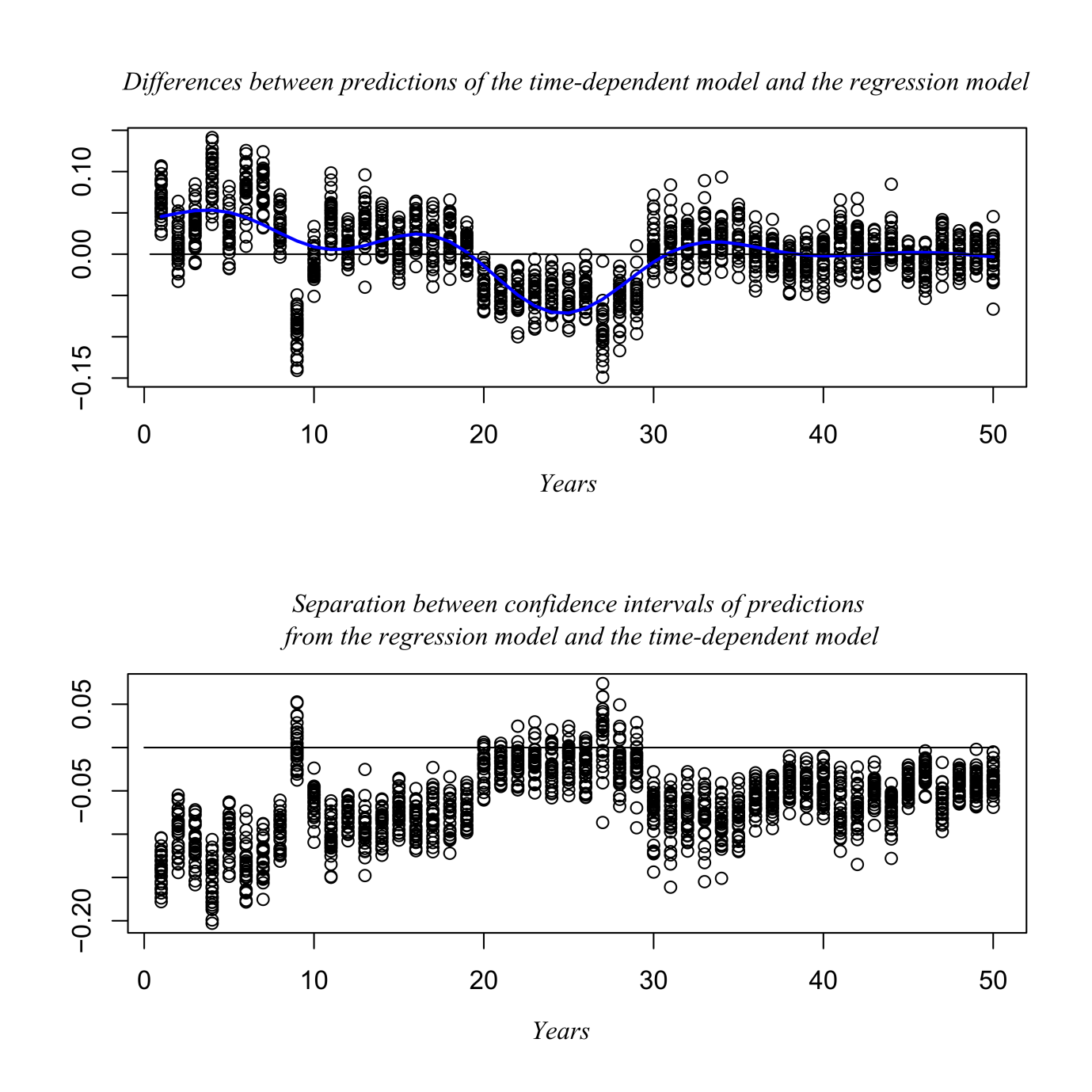


References specific to the Appendices

Bunge, J., Woodard, L., Böhning, D., Foster, J. A., Connolly, S., & Allen, H. K. (2012) Estimating population diversity with CatchAll. *Bioinformatics*, 28, 1045-1047.

Stasinopoulos, D. M., Rigby R.A. (2007) Generalized additive models for location scale

and shape (GAMLSS) in R. *Journal of Statistical Software* 23.
